# Sexual dysfunction in chemsex users - research report

**DOI:** 10.1192/j.eurpsy.2023.2336

**Published:** 2023-07-19

**Authors:** R. M. Kowalczyk, A. Stola

**Affiliations:** 1 Psychology; 2 Andrzej Frycz Modrzewski Krakow University, Krakow, Poland

## Abstract

**Introduction:**

Epidemiology and etiology of sexual disorders and problems have been described in detail for heterosexual men. In the case of MSM, scientific studies are much more limited, and the presented frequencies depend largely on the methodology of the study and the population covered by the study (socio-cultural differences, influence of minority stress). For chemsex users, the data is even more enigmatic; the subchapter presents data from the research currently conducted in Poland that has not yet been published.Most studies in the recent past have focused on assessing the risk of HIV transmission, sexual dysfunction associated with diagnosis, and the use of antiretroviral drugs. Only recently have been published papers, though still few, assessing the frequency of specific sexual dysfunctions among MSM, which are included as a whole group. It should be emphasized that there is currently no conclusive evidence to suggest a different diagnostic or therapeutic approach for sexual dysfunction in MSM patients.

**Objectives:**

Most studies in the recent past have focused on assessing the risk of HIV transmission, sexual dysfunction associated with diagnosis, and the use of antiretroviral drugs. Only recently have been published papers, though still few, assessing the frequency of specific sexual dysfunctions among MSM, which are included as a whole group. It should be emphasized that there is currently no conclusive evidence to suggest a different diagnostic or therapeutic approach for sexual dysfunction in MSM patients. The aim of the presented report is to supplement the knowledge about the MSM population of Chemsex users.

**Methods:**

A focus group of 60 men aged 18-50 split was assembledinto four groups depending on the type of sexual contact (ChemSex non-ChemSex) and HIV +/-. The focus data was supplemented with research tools: a questionnaire containing 75 questions - elements of medical and sexological interview, and a standardized scale: The quality of life questionnaire in relation to sex - Male (Polish version of the SQoL-M), Premature ejaculation questionnaire and the International Index of Erectile Function (IIEF).

**Results:**

The results are being analyzed. The full report will be presented at the conference.

**Conclusions:**

The aim of the project is to assess the quality of sexual life and to examine the frequency of sexual dysfunction in the MSM population, active Chemsex users and the frequency of sexual contacts, the number of sexual partners, perceived sexual needs and satisfaction, as well as to determine the psychosocial / medical factors influencing the occurrence of sexual dysfunction.

Supplementing knowledge in this field will allow for more effective therapeutic effects.

**Disclosure of Interest:**

None Declared

